# Organ-Specific Oxidative Events under Restrictive Versus Full Reperfusion Following Hemorrhagic Traumatic Shock in Rats

**DOI:** 10.3390/molecules23092195

**Published:** 2018-08-30

**Authors:** Carina Penzenstadler, Anna Zifko, Mohammad Jafarmadar, Janin Schulte, Joachim Struck, Michaela Stainer, Andrey Kozlov, Soheyl Bahrami

**Affiliations:** 1 Ludwig Boltzmann Institute for Experimental and Clinical Traumatology, AUVA Research Center for Traumatology, 1200 Vienna, Austria; carina.penzenstadler@gmail.com (C.P.); Anna.Zifko@trauma.lbg.ac.at (A.Z.); Mohammad.Jafarmadar@trauma.lbg.ac.at (M.J.); Michaela.Stainer@trauma.lbg.ac.at (M.S.); Andrey.Kozlov@trauma.lbg.ac.at (A.K.); 2 Thermo Fisher Scientific, BRAHMS Biomarkers, Research Department, 16761 Hennigsdorf, Germany; janin.schulte@mail.de (J.S.); jstruck@adrenomed.com (J.S.)

**Keywords:** reactive oxygen species, peroxidation, ischemia, reperfusion injury, organ failure

## Abstract

Background aim: Reperfusion after hemorrhagic traumatic shock (HTS) is often associated with complications that are partly ascribed to the formation of reactive oxygen species (ROS). The aim of our study was to compare the effects of restrictive reperfusion (RR) to rapid full reperfusion (FR) on ROS formation and/or oxidative events. Materials and methods: Anesthetized male rats were randomly subjected to HTS followed by FR (75 mL/kg/h) or RR (30 mL/kg/h for 40 min, followed by 75 mL/kg/h) with Ringer’s solution (n = 8/group). Compartment-specific ROS formation was determined by infusion of ROS scavenger 1-hydroxy-3-carboxy-2,2,5,5-tetramethyl-pyrrolidine hydrochloride (CP-H) during resuscitation, followed by electron paramagnetic resonance spectroscopy. Sham-operated animals (n = 8) served as controls. The experiment was terminated 100 min post-shock. Results: Mean arterial pressure was significantly higher in the FR compared to the RR group during early reperfusion. Only RR animals, not FR animals, showed significantly higher ROS concentrations in erythrocytes (1951 ± 420 vs. 724 ± 75 AU) and in liver (474 ± 57 vs. 261 ± 21 AU) compared to sham controls. This was accompanied by elevated alanine aminotransferase and creatinine levels in RR animals compared to both shams and FR animals, while lipid peroxidation products (thiobarbituric acid reactive substances) were significantly increased only in the kidney in the FR group (*p* < 0.05). RR animals showed significantly higher plasma peroxiredoxin-4 values when compared to the FR group (20 ± 2 vs. 14 ± 0.5 RLU). Conclusion: Restrictive reperfusion after HTS is associated with increased ROS formation in erythrocytes and liver compared to sham controls. Moreover, the restrictive reperfusion is associated with a more pronounced injury to the liver and kidney, which is likely mediated by other than lipid peroxidation process and/or oxidative stress reactions.

## 1. Introduction

Hemorrhagic traumatic shock (HTS) is a major cause of death in people, regardless of demographics. Mortality is directly associated with severe blood loss and coagulopathy as well as secondary complications like infections and/or multiple organ failure (MOF) [1]. The pathophysiology of HTS is complex and still not completely understood. Briefly, the massive blood loss causes hypotension and reduced tissue perfusion with a subsequent imbalance of oxygen delivery and oxygen consumption [2]. Therefore, effective hemorrhage control and fluid resuscitation are the main therapeutic approaches to improve survival. There is an ongoing discussion about optimal resuscitation-on the one hand, about reperfusion strategies (rapid, delayed, hypotensive); and on the other hand, about the type of fluids, such as crystalloids, colloids and blood [3]. However, restoration of blood pressure and tissue perfusion by rapid infusion of large volumes of fluids can be detrimental. At least under uncontrolled bleeding, the so-called full reperfusion (FR) can promote rebleeding, dislodgment of blood clots, and additional injury to the formerly ischemic tissue [1]. In addition, reoxygenation of the formerly hypoxic cells is associated with activation of leukocytes and release of proinflammatory proteins as well as enhanced formation of reactive oxygen species (ROS). In general, ROS such as superoxide, hydrogen peroxide and hydroxyl anions are responsible for damage to molecules such as proteins, lipids, and DNA. While under physiological conditions damaging effects are counteracted by a number of antioxidants during reperfusion, these natural defenses may be overwhelmed, and ROS can lead to additional tissue damage [4,5]. Therefore, resuscitation strategies that minimize the release of ROS are desirable. Restrictive reperfusion (RR), which allows restoration of blood circulation with a modest increase in blood pressure, is attractive for prehospital settings. Using RR, different studies have described beneficial effects, such as decreased blood loss, better splanchnic perfusion, reduced inflammation, and decreased apoptotic cell death [6,7,8]. In addition, Zifko et al. showed that ROS formation under restrictive reperfusion does not affect organ function [9].

The aim of the present study was to investigate the organ-specific ROS formation during restrictive reperfusion in comparison to full reperfusion after HTS and its potential pathophysiological relevance.

## 2. Material and Methods

### 2.1. Animals

Twenty-four male Sprague Dawley rats (390–490 g; Animal Research Laboratories, Himberg, Austria) were kept under controlled standard conditions with free access to standard laboratory rodent food and water during an adaptation period of at least 7 days before use in this study. All experimental procedures were in accordance with the Guide for the Care and Use of Laboratory Animals as defined by the National Institutes of Health and were approved by the Animal Protocol Review Board of the city government of Vienna, Austria (MA58/6294/09/10).

### 2.2. Anesthesia and Instrumentation

The rats were deeply sedated in a preflooded box with 3% isoflurane for 1–2 min. To maintain surgical depth anesthesia during the instrumentation period, 1% isoflurane was administered via mask. The animals were positioned on a temperature-controlled surgical board (36–37 °C) during the entire experiment. The left femoral vein was cannulated with a silicone catheter and connected to a three-way stopcock for intravenous (i.v.) application of anesthetics, radical scavenger, and fluid treatment. S-ketamine was applied i.v. at a rate of 60 mg/kg/h in combination with xylazine (2.5 mg/kg) intramuscularly. During shock, the S-ketamine rate was reduced to 30 mg/kg/h. Left femoral artery was cannulated for measurement of hemodynamic parameters and withdrawal of blood.

### 2.3. Hemorrhagic Traumatic Shock Model

**Figure 1:**
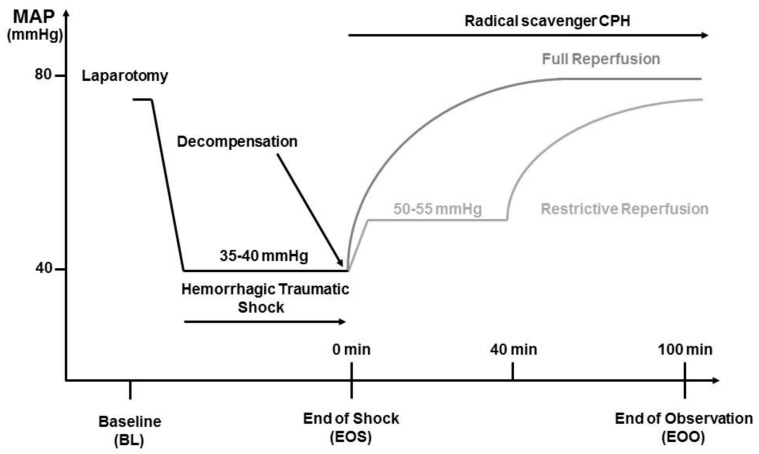
Experimental set-up of hemorrhagic traumatic shock (HTS) and following reperfusion strategies. Rats were subjected to HTS, including a midline laparotomy and hemorrhage, to a mean arterial blood pressure (MAP) of 35–40 mmHg until reversible decompensation. Then, the animals received a restricted reperfusion (30 mL/kg/h for 40 min to achieve a MAP of 50–55 mmHg) or a full reperfusion (75 mL/kg/h) with Ringer’s solution until a total reperfusion volume of four times shed blood plus 20 mL was achieved. The radical scavenger CP-H (1-hydroxy-3-carboxy-2.2.5.5-tetramethylpyrrolidine) was continuously infused during the entire reperfusion time of 100 min. Sham-operated animals served as controls (not shown). Samples were taken at baseline (BL), end of shock (EOS), and end of observation (EOO).

The experimental procedure is illustrated in Figure 1. After instrumentation of the vessels, hemodynamic parameters were allowed to stabilize for at least 5 min before baseline sampling and the initiation of HTS. Heart rate (HR) and mean arterial blood pressure (MAP) were monitored during the entire time of the experiment using PowerLab software system (ADInstruments Ltd., Oxford, UK). To simulate a trauma, a midline laparotomy was performed immediately before the onset of hemorrhage. The incision was covered with saline-soaked gauze and closed after 20 min. Hemorrhage was initiated by manual blood withdrawal over five minutes until a MAP of 35 to 40 mmHg was reached. This pressure was maintained until the onset of decompensation (after 50–90 min), a condition characterized by reversible blood pressure collapse that can be prevented by repeated and obligatory fluid infusion.

### 2.4. Reperfusion Strategies

At the time of decompensation, animals were randomly assigned to two different groups. Animals assigned to the restrictive reperfusion group (n = 8) received Ringer’s solution (Fresenius Kabi, Graz, Austria) at a rate of 30 mL/kg/h (as needed) to maintain MAP between 50 and 55 mmHg for the first 40 min. Resuscitation was then continued using a rate of 75 mL/kg/h (four times the shed blood volume). Animals assigned to the full reperfusion group (n = 8) received Ringer solution at a rate of 75 mL/kg/h (four times the shed blood volume over 60 min). Thereafter, infusion was continued at a rate of 30 mL/kg/h for 40 min. Sham-operated animals (n = 8) were subjected to the same anesthesia and instrumentation protocol as shock-operated animals but without undergoing HTS and reperfusion. The experiment was terminated at 100 min of resuscitation.

### 2.5. ROS Scavenging

For detection of superoxide and peroxynitrite radicals, the membrane permeable radical scavenger CP-H (1-hydroxy-3-carboxy-2.2.5.5-tetramethylpyrrolidine, Noxygen Science Transfer & Diagnostics, Germany) was administered immediately as a bolus of 9.0 mg/kg intravenously at the onset of reperfusion, followed by a continuous infusion at the rate of 0.225 mg/kg/h during the entire resuscitation time of 100 min. The CP-H was infused via a separate line and infusion pump, thereby providing the same amounts/rate of CPH for each animal throughout the experiment independent of the fluids infusion rate. The compound CP-H was dissolved immediately before use in 0.9% NaCl with 20 µM Desferal to a concentration of 5 mg/mL.

### 2.6. Blood and Organ Sampling

Blood samples were obtained at baseline (BL), end of shock (EOS), and at the end of observation (EOO). At the end of experiment, animals were sacrificed and organs (kidney, spleen, liver, heart, lung, ileum, and colon) were harvested.

For ROS detection in red blood cells (RBC) and plasma, 3 mL of heparinized whole blood were centrifuged (Centrifuge 5415 R, Eppendorf, Germany) at 7200 rpm for 15 min at room temperature. The plasma supernatant and the remaining red blood cells, after removal of the buffy coat, were aspirated into two separate syringes and frozen in liquid nitrogen. Furthermore, one aliquot of each organ was placed in a 1-mL syringe and quickly frozen in liquid nitrogen. The frozen samples were pressed out of the syringes and stored in tubes (Cryos, Greiner, Germany) at −80 °C until EPR measurement.

The remaining tissue aliquots were frozen in liquid nitrogen and kept at −80 °C for thiobarbituric acid reactive substances and myeloperoxidase analysis.

### 2.7. Blood Analysis

Heparinized arterial blood was used to measure pH, base excess (ABEc), lactate, carbon dioxide (CO_2_), and oxygen (O_2_) partial pressure using an ABL 625 System blood gas analyzer (Radiometer Medical A/S, Copenhagen, Denmark). Heparinized plasma samples were used for measurement of creatine kinase (CK), lactate dehydrogenase (LDH), alanine aminotransferase (ALT), creatinine (Crea), and urea with an automatic analyzer (Cobas c111, Roche Diagnostics, Austria). Plasma samples were stored at −80 °C. Blood cell counts (hematocrit, erythrocytes, platelets, and leukocytes) were determined using a CELL-DYN 1300 instrument (Abbott, Vienna, Austria).

### 2.8. Electron Paramagnetic Resonance (EPR) Spectroscopy

Electron paramagnetic resonance (EPR) spectroscopy was used to determine the ROS production in frozen samples of animals receiving CP-H. CP-H is oxidized by reaction with ROS to form the stable 3-CP compound, which is detectable by EPR. The spectra were recorded at liquid nitrogen temperature with an EPR spectrometer (MiniScope MS200, Magnatech, Berlin, Germany). The following settings were used: microwave power 1 mW, MW attenuation 20 dB, frequency 9.5 GHz, modulation amplitude 5 G, field center 3361 G, field sweep 97 G, and sweep time 120 s.

### 2.9. Oxidative Stress Parameters

Thiobarbituric acid reactive substances (TBARS) concentration was determined using QuantiChromTM TBARS assay kit (BioAssay Systems, Hayward, CA, USA). The analysis was performed according to the manufacturer’s instructions by addition of 3 µM butylhydroxytoluol and 20 µM Desferal to the samples before homogenization to prevent further oxidation. TBARS were measured using a POLARstar Omega fluorescence microplate reader (BMG LABTECH, Ortenberg, Germany).

The antioxidative enzyme peroxiredoxin-4 (Prx-4) was measured in a sandwich chemoluminescence immunoassay using two antibodies directed against a peptide that corresponds to amino acids 42 to 54 of mouse Prx-4. Homology to amino acids 41 to 53 of rat Prx-4 was confirmed. The assay procedure has been described earlier [10]. Liver samples were immediately frozen in liquid nitrogen, mechanically pulverized and lysed by sonication in HEPES–sucrose buffer in order to extract intracellular protein. The protein concentration was determined using bicinchoninic acid assay (Thermo Scientific Pierce, Rockford, IL, USA). Results are given as relative luminescence units (RLU) per mg of protein.

### 2.10. Lung Damage Parameters

Neutrophil infiltration of the lung was determined by measuring myeloperoxidase (MPO) activity by a slight modification of the method described by Mullane et al. [11]. Briefly, approximately 100 mg of thawed lung tissue was placed in 1 mL 20 mM potassium phosphate buffer (pH 6.0) with 0.5% hexadectyltrimethylammonium bromide. The tissue was homogenized, sonicated, incubated at 60 °C for 2 h to remove pseudoperoxidases, and centrifuged for 15 min at 13,000× *g*. The supernatant was assayed for MPO activity using kinetic spectrophotometric reaction with *o*-dianisidine dihydrochloride (Sigma Chemicals Co., St Louis, MO, USA), detected at 450 nm (Spectrophotometer SLT-Spectra, Tecan, Austria).

Pulmonary liquid infiltration was measured by determining the wet versus dry weight ratio of the lower left lobe of the lung after drying at 110 °C in a vacuum oven for 48 h.

### 2.11. Statistical Analysis

All parameters were tested for normality (Kolmogorov–Smirnov test) prior to analysis. Statistical evaluation of data was performed by one-way ANOVA followed by Tukey’s post-test for parametric distribution and Kruskal–Wallis followed by Dunn’s post-test for nonparametric distribution. Differences were considered significant if *p* < 0.05. For all statistical analyses, GraphPad Prism 5.01 (GraphPad Software, San Diego, CA, USA) was used. Data are represented as mean ± SEM.

## 3. Results

### 3.1. HTS and Resuscitation Results in Reproducible Alteration of Hemodynamics and Shock Parameters

Our shock model revealed reproducibility in the amount of shed blood (7% of body weight) and shock duration and did not differ significantly between the two shock groups (Table 1). As a consequence, hematocrit, erythrocyte, and platelet counts dropped equally in both reperfusion groups and did not show any differences at the EOO. In addition, all parameters were significantly lower compared to sham-operated animals (*p* < 0.001). Similarly, leucocyte counts decreased significantly due to hemorrhage and reperfusion in RR and FR group (*p* < 0.01 and *p* < 0.002, respectively) but without any difference to sham-operated animals.

**Figure 2:**
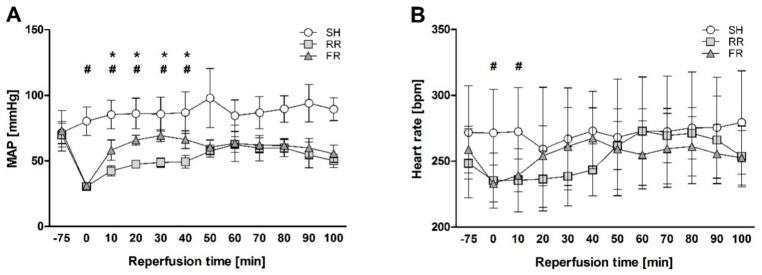
Chronological sequence of mean (**A**) arterial blood pressure (MAP) and (**B**) heart rate in sham (SH), restricted reperfusion (RR), and full reperfusion (FR) group during the entire experiment. MAP and heart rate were measured at baseline (−75 min), end of shock (0 min), and during reperfusion until the end of observation (100 min). Data are presented as mean ± SEM, n = 8 in each group, * indicates significance between RR and FR group, # indicates significance between sham and shock, *p* < 0.05.

Mean arterial blood pressure, heart rate and body temperature (BT) were monitored during the entire experiment. The MAP did not show any differences between the two shock groups at baseline and at EOS. From EOS onwards, MAP differed significantly between the two shock groups during the early phase of reperfusion. In the RR group, MAP was significantly lower during the first 40 min reperfusion as required by the study protocol (*p* < 0.001). Afterwards, blood pressure was nearly identical (showed a similar trend) in both shock groups (Figure 2). As a consequence of hemorrhage and decrease in blood pressure, HR dropped significantly in both shock groups at EOS and during the first 10 min of reperfusion. However, HR did not differ between the groups during the remaining reperfusion time (Figure 2). BT did not differ between the groups at any point of time (data not shown).

According to our shock model, arterial blood gas sampling showed metabolic acidosis at the end of shock in both groups. The pH and base excess decreased, while carbon dioxide partial pressure (pCO_2_) decreased and oxygen partial pressure (pO_2_) increased due to respiratory compensation. In addition, as an indicator of tissue minor perfusion, lactate levels also increased at the time of decompensation. There were no differences between the two shock groups, but both groups were significantly different to the sham. However, at the end of observation, resuscitation with Ringer’s solution resulted in a decrease of lactate to baseline levels, whereas pH, ABEc, and pO_2_ remain unchanged (Table 2).

### 3.2. Compartment-Specific Changes in ROS Formation and Oxidative Events after Hemorrhage and Resuscitation

**Figure 3:**
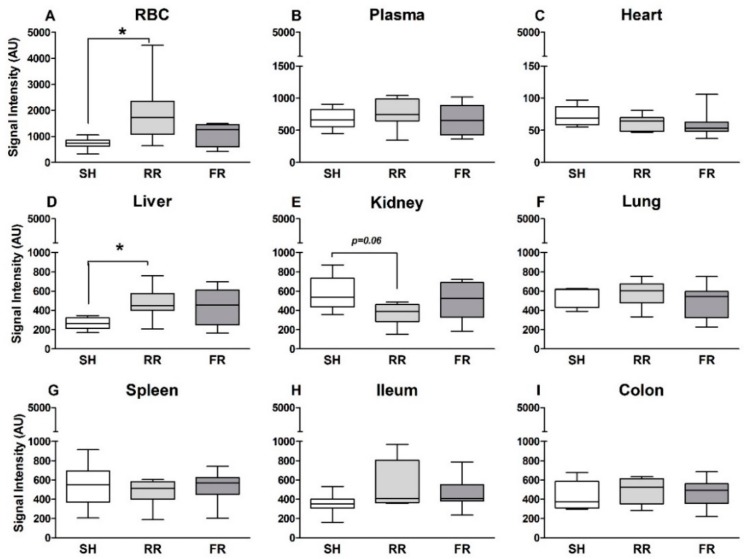
Compartment-specific ROS formation (A – I) in animals subjected to HTS and restricted reperfusion (RR) or full reperfusion (FR) and sham controls (SH) at the end of experiment. RBC = red blood cells. Data are presented as median, boxes indicate 25th and 75th percentiles, whiskers represent the range, n = 8 in each group, * *p* < 0.05.

EPR analysis revealed organ-specific ROS formation. The highest levels were found in red blood cells (RBC) (SH: 724 ± 75 AU; RR: 1951 ± 420 AU; FR: 1095 ± 154 AU) and the lowest in the heart (SH: 72 ± 6 AU; RR: 62 ± 4 AU; FR: 59 ± 7 AU). Only restrictive reperfusion resulted in a significant increase in ROS formation in RBCs and in liver (*p* < 0.05) when compared to sham controls (Figure 3A,D). In the kidney, ROS levels were lower in the RR group than in the sham group but did not reach significance (Figure 3E)*.* Hemorrhage and reperfusion did not affect ROS formation in plasma heart, lung, spleen, ileum, and colon compared to sham animals (Figure 3B,C,F–I). Comparing the two shock groups, there was no difference in ROS formation between the two reperfusion regimens in all different compartments analyzed (Figure 3A–I).

**Figure 4:**
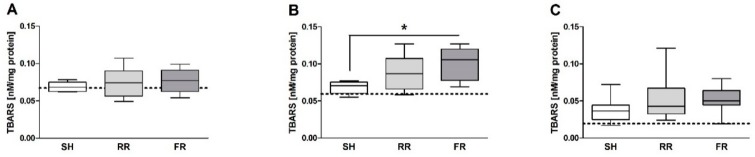
Thiobarbituric acid reactive substances (TBARS) formation in (**A**) liver, (**B**) kidney, and (**C**) ileum in sham-operated (SH) and animals subjected to HTS and resuscitation. RR = restricted reperfusion, FR = full reperfusion. TBARS formation was determined at the end of observation. Data are presented as median, boxes indicate 25th and 75th percentiles, whiskers represent the range, n = 8 in each group, * *p* < 0.05. The dash lines represent the average laboratory control values obtained in 5 healthy animals.

Thiobarbituric acid reactive substances, an end product of lipid peroxidation, did not differ between groups in liver and ileum (Figure 4A,C). However, in the kidney, TBARS were significantly higher in the FR group when compared to sham group (Figure 4B)*.*

**Figure 5:**
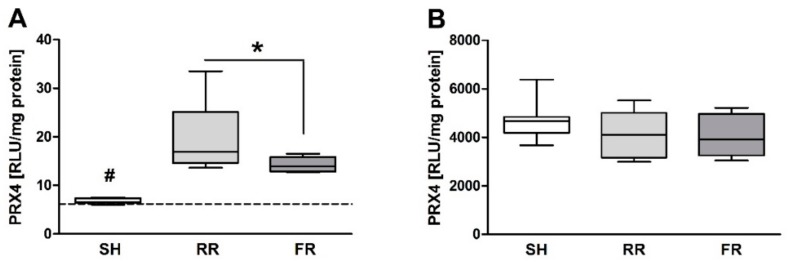
Oxidative stress measured by the antioxidative enzyme peroxyredoxin-4 (Prx-4) in plasma (**A**) and liver (**B**) in sham-operated animals (SH) and animals subjected to HTS and resuscitation. RR = restricted reperfusion, FR = full reperfusion. Data are presented as median, boxes indicate 25th and 75th percentiles, whiskers represent the range, n = 8 in each group, * indicates significance at EOO between RR versus FR, # indicates significance at EOO between SH versus both reperfusion groups, *p* < 0.05. The dash line represents the average laboratory control value obtained in five healthy animals.

Peroxiredoxin-4 (Prx-4) is an antioxidative enzyme that is increased during oxidative stress. Prx-4 in plasma increased in both shock groups (RR: *p* < 0.001; FR: *p* < 0.01) at the end of observation when compared to sham controls (Figure 5A). Furthermore, the RR group displayed significantly higher plasma levels *(p* < 0.05) of Prx-4 when compared to the FR group (Figure 5A). However, Prx-4 concentrations in liver did not differ between the groups (Figure 5B).

### 3.3. Cell and Organ Injury

**Figure 6:**
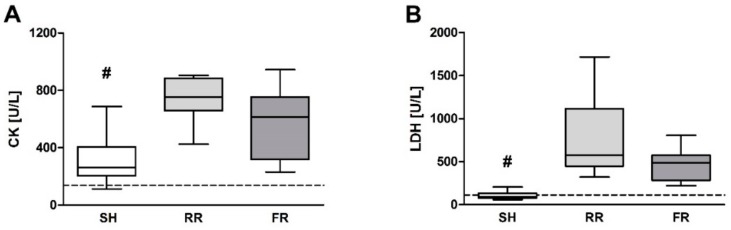
Cellular injury in sham-operated animals (SH) and in restricted reperfusion (RR) and full reperfusion (FR) group. (**A)** Plasma creatine kinase (CK) and (**B**) lactate dehydrogenase (LDH) were measured at baseline (dotted line) and end of observation. Data are presented as median, boxes indicate 25th and 75th percentiles, whiskers represent the range, n = 8 in each group, # indicates significance at EOO between SH versus both reperfusion groups, *p* < 0.05. The dash lines represent the average laboratory control values obtained in five healthy animals.

At the end of observation, the hemorrhage- and reperfusion-induced cellular injury assessed by creatine kinase (CK) and lactate dehydrogenase (LDH) were significantly enhanced in the RR group compared to sham animals, with no difference between the two reperfusion regimens (Figure 6A,B).

**Figure 7:**
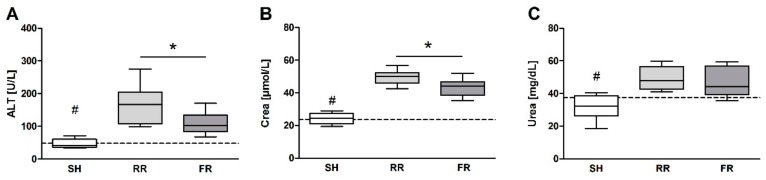
Alteration of organ function in sham-operated animals (SH) and in restricted reperfusion (RR) and full reperfusion (FR) group. Plasma alanine aminotransferase (ALT), creatinine (Crea), and urea were measured at baseline (dotted line) and end of observation (**A**–**C**). Data are presented as median, boxes indicate 25th and 75th percentiles, whiskers represent the range, n = 8 in each group, * indicates significance at EOO between RR versus FR, # indicates significance at EOO between SH versus both reperfusion groups, *p* < 0.05. The dash lines represent the average laboratory control values obtained in five healthy animals.

HTS followed by reperfusion resulted in a significant increase in ALT, Crea and urea at EOO when compared to sham (*p* < 0.05). In addition, Crea and ALT values were significantly higher in the RR group when compared to the FR group (Figure 7A–C).

**Figure 8:**
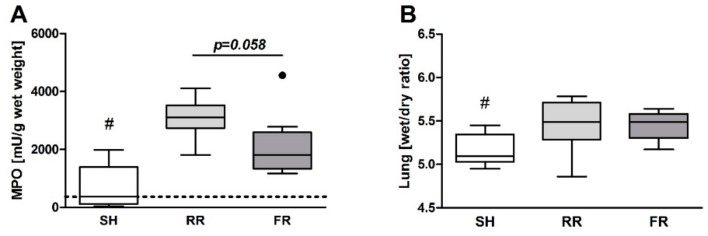
(**A**) Migration of neutrophils and (**B**) pulmonary liquid infiltration into lungs in sham-operated animals (SH) and animals subjected to HTS and resuscitation. RR = restricted reperfusion, FR = full reperfusion. (**A**) Myeloperoxidase activity (MPO) and (**B**) wet/dry ratio was measured in lung tissue at end of observation. Data are presented as median, boxes indicate 25th and 75th percentiles, whiskers represent the range, n = 8 in each group, # *p* < 0.05 versus other groups. The dash line represents the average laboratory control value obtained in five healthy animals.

Myeloperoxidase (MPO), reflecting the migration of neutrophils in lung tissue, was enhanced in both shock groups at the end of observation, reaching significance level (*p* < 0.001) in the RR group compared to sham (Figure 8A). The wet/dry weight ratio reflecting liquid infiltration into the lung tissue increased in both groups (*p* < 0.05 vs. sham) with no difference between groups (Figure 8).

## 4. Discussion

It has been widely documented that damage to the tissue mediated by ischemia/reperfusion occurs during reperfusion phase due to the activation of oxidative stress, a process initiated by one electron reduction of molecular oxygen delivered to tissues during reperfusion. In contrast to normoxic tissue, reoxigenation of ischemic tissue is accompanied by oxidative stress because a number of pro-oxidant molecules are generated during ischemic phase, such as xanthine oxidase [12] or free iron [13]. Hemorrhagic shock is associated with a global ischemia, and acute organ failure is a frequent complication after reperfusion. Current guidelines recommend a rapid restoration of blood pressure with subsequent restoration of tissue oxygenation. However, a rapid reperfusion/oxygenation is supposed to be accompanied by excessive ROS release and subsequent additional cell and tissue damage. Our present study does not confirm this assumption. By infusing the radical scavenger CP-H followed by EPR analysis, we determined that radicals such as superoxide and peroxynitrite were formed in individual compartments during the reperfusion period after hemorrhagic/traumatic shock. Although it is not yet clearly defined what CP-H measures, the assay itself has the advantage that tissue samples are measured frozen without being necessarily processed. As the assay is standardized and reproducible, the relative changes and differences observed are shock- and reperfusion-related. Our study showed organ-specific changes in ROS formation following HTS, which was influenced by the mode of reperfusion. Compared to sham controls, the restrictive reperfusion (RR group) was associated with enhanced ROS formation in RBCs and in the liver; however, this was not the case for rapid full reperfusion (FR group). In other compartments, such as heart, lung, spleen, intestine, and plasma ROS, formation was similar in all three groups—sham, RR, and FR. 

Our results showed trendwise lower ROS formation in kidney in the RR group compared to sham. A possible explanation for the reduced accumulating ROS in the kidney could be that during RR, less oxygen radicals are formed because of insufficient renal oxygenation. Our previous study [9] has shown that a decline in perfusion is associated with a decrease in both 3-CP and CP-H-derived 3-CP concentration in kidney. It may also be that the kidney has sufficient antioxidative capacity to protect the organ from damage caused as a consequence of minor perfusion and ROS formation. Thus, interpretation of the kidney concentration of ROS in the RR group is limited. More importantly, even an instant reperfusion (FR group) did not affect ROS formation when compared to the sham controls. In addition, the elevated TBARS noted in the kidney in the FR group did not differ to the RR group. Finally, the renal dysfunction, as assessed by the plasma creatinine concentration, was significantly ameliorated in FR compared to the RR group. Our results are in line with the report by Legrand et al. [14], which showed that rapid correction of the MAP (80 mmHg) with saline restored renal blood flow and creatinine clearance in comparison to a low MAP resuscitation (40 mmHg). This suggests that upon RR, kidney—as an organ with higher perfusion rate—suffers more from reduced blood flow than from hypoxia. Consequently, elevated levels of creatinine observed in RR could be due to reduced perfusion rate rather than due to kidney damage. In fact, it has been shown that glomerular filtration rate and renal plasma flow decreased proportionally upon lowering MAP [15].

Liver cells contain high levels of mitochondria, which are believed to be the main source for ROS formation. Therefore, it is not surprising that ROS formation is markedly increased in liver after HTS and reperfusion in both reperfusion groups, reaching significance in the RR group. In line with this, HTS and reperfusion induced liver injury, as reflected by an increase in ALT levels. Importantly, this increase was significantly higher in the RR group compared to the FR group, demonstrating that prolonged hypoxia associated with enhanced ROS formation has an amplifying influence on HTS-induced liver injury. Two earlier studies implicating the same restricted reperfusion model have shown no increased ROS levels in liver tissue after 40 min of restricted reperfusion by EPR measurement [9,16]. The study by Duvingeau et al. showed no increased ROS levels in the liver after 3 h of reperfusion. The present experiment was terminated 100 min after reperfusion, an intermediate time point, indicating a temporal transition of ROS levels during reperfusion. However, we observed elevated TBARS concentrations in the liver. It is reasonable that the antioxidative capacity of the organism is still efficient enough in neutralizing ROS at this early time point. However, at later time points, this capacity may be exhausted and further tissue damage could occur due to oxidative stress.

We detected the highest ROS formation in the circulation, namely in the red blood cells (RBC) and not in organs. As mentioned above, the main ROS source in cells is mitochondria. Despite lacking mitochondria, ROS are continuously produced in RBCs due to the oxygen carrier capacity of hemoglobin and high O_2_ tension in arterial blood [17]. The increased ROS formation in the RR group is most likely due to increased auto-oxidation of hemoglobin. Scavenging superoxide radicals by CP-H does not affect the ROS generated by HTS and reperfusion. Thus, one may assume that radicals other than superoxide, such as peroxynitrite, are involved. Puppo and Halliwell estimated that oxyhemoglobin and methemoglobin caused formation of OH^−^ radicals from H_2_O_2_ [18]. However, in the present study, we did not specify the amounts of OH^−^ radical. This remains to be characterized in further studies.

Oxidative stress is defined as an imbalance between ROS formation and the antioxidative capacity of the organism. We evaluated plasma levels of the antioxidative enzyme peroxiredoxin-4 (Prx-4), which is notably the only secreted isoform of the Prx family in humans and animals [19,20]. Prx-4 is augmented during oxidative stress [21]. In our study, Prx-4 levels were elevated in plasma during reperfusion with the RR group, showing significantly higher Prx-4 levels than the FR group. In general, oxidative stress is tissue-dependent, and processes in plasma and RBCs can differ. Prx-1 to Prx-6 may be expressed in different tissues and cell compartments where they exert their antioxidant and other functions. Prx-4 is ubiquitously expressed with highest expression in the pancreas, liver, and heart. However, in the present study, prx4 levels did not differ between sham and shock groups. In RBCs, Prx-2 (not Prx-4) is one of the most prominent antioxidants [22]. There is not necessarily a link between high ROS content in RBCs and increased Prx-4 levels in plasma. Prx4 secretion could lead to a loss of intracellular or membrane-bound Prx4, which would result in a changed capacity to remove H_2_O_2_, to regulate redox mechanisms or to participate in signaling pathways in the cellular vicinity. Thus, we speculate that prolonged minor perfusion that occurs during RR leads to more oxidative stress than fast reperfusion, resulting in enhanced release of prx4.

Numerous studies have suggested that oxidative stress is also involved in the pathogenesis of cardiovascular diseases. Abbasi et al. have given evidence that elevated Prx4 levels are associated with higher risk of incident cardiovascular disease [23]. However, in our study, direct RONS measurement by EPR showed the lowest ROS levels in the heart, maybe due to the fact that the heart—as the most vital organ—is still perfused enough during shock and reperfusion. This circumstance is also displayed by plasma CK levels, which increased equally in both reperfusion groups when compared to sham.

Despite equal ROS formation, neutrophil infiltration into the lungs was more pronounced in the FR group. Nevertheless, lung edema after HTS and reperfusion did not differ between the RR and FR groups. One may assume that the HTS-induced lung permeability changes observed in the present study are not directly related to the ROS formation in the lung. In this respect, it is important to note that CP-H treatment according to our protocol scavenges the ROS that are potentially formed in organs.

In the present study, TBARS concentration in liver and kidney were not in line with the findings of direct ROS formation detected in organs. While ROS formation in liver was significantly higher in the RR group compared to sham animals, TBARS did not differ between the groups. Similarly, lower ROS formation in the kidney was paralleled by elevated TBARS. TBARS are the product of lipid peroxidation; their accumulation indicates the damage to biomembranes. The absence of a coincidence between liver damage marker (ALT) and TBARS indicate that lipid peroxidation was probably not the leading process resulting in increased ALT release in the RR group. Recently, an activation of a number of signaling cascades, such as hypoxia inducible factor (HIF) and vascular endothelial factor (VEGF), has been suggested to be key mechanisms in the ROS action in hypoxia reperfusion [24,25].

Considering the gut as a possible source in the development of MOF [26], we determined oxidative events in the ileum by assessing ROS formation and TBARS. The RR regime has been associated with a delay in MAP recovery (MAP significantly lower in the RR group than in FR within the first 40 min), most likely paralleled by lower perfusion rate of organs in the RR group compared to the FR group. We found no significant differences between the shock and the sham groups in the present study. The lack of adverse effect on ileum might be due to the higher resistance of rats to the under perfusion. However, ROS and TBARS levels showed an ascending trend when compared to sham, indicating the susceptibility of this organ to HTS and reperfusion.

Our data suggest that only the restrictive reperfusion after HTS is associated with increased ROS formation in erythrocytes and liver compared to the sham controls. Moreover, compared to the full reperfusion, the restrictive reperfusion is associated with more pronounced liver and kidney injury. Possible contribution of ROS to liver damage is most likely due to ROS-mediated signaling rather than due to oxidative damage of biomolecules, e.g., by lipid peroxidation. Increased levels of creatinine in blood are due to low rate of kidney perfusion rather than kidney tissue damage. Increased release of the antioxidant enzyme peroxiredoxin-4 into plasma can be a good marker of enhanced oxidative stress due to prolonged minor perfusion.

## Figures and Tables

**Table 1:** Body weight, shock duration, and shed blood in sham-operated animals and animals subjected to HTS and resuscitation.

Group	Body Weight (kg)	Shock Duration (min)	Shed Blood (%)	Shed Blood (mL)
SH	0.42 ± 0.01	78.38 ± 2.21	8.40 ± 0.84	2.5 ± 0.29
RR	0.43 ± 0.01	78.75 ± 2.28	45.05 ± 1.58 ^#^	13.64 ± 0.67 ^#^
FR	0.43 ± 0.01	85.00 ± 2.75	43.51 ± 2.37 ^#^	13.18 ± 0.75 ^#^

SH = sham-operated animals, RR = restricted reperfusion, FR = full reperfusion. Shed blood in SH due to blood sampling. n = 8 animals in each group. Data are presented as mean ± SEM, ^#^ indicates significance versus sham, *p* < 0.05.

**Table 2:** Alteration of shock parameters in sham-operated animals and animals subjected to HTS and resuscitation.

Group	Time Point	pH	ABEc, mmol/L	Lactate, mg/dL	pO_2_, mmHg	pCO_2_, mmHg
SH	BL	7.35 ± 0.01	0.13 ± 0.61	4.8 ± 0.6	62.8 ± 2.6	47.3 ± 1.3
	EOS					
	EOO	7.32 ± 0.01	−2.35 ± 0.85	5.3 ± 0.6	69.1 ± 2.2	45.6 ± 1.3
RR	BL	7.34 ± 0.01	0.48 ± 1.07	4.1 ± 0.4	72.2 ± 10.4	48.8 ± 2.5
	EOS	7.21 ± 0.01 *	−14.16 ± 0.65 *	46.4 ± 3.0 *	90.2 ± 3.4 *	31.6 ± 1.7 *
	EOO	7.16 ± 0.05 *^,#^	−13.35 ± 1.32 *^,#^	9.6 ± 1.3 ^†,#^	87.0 ± 6.5 *	41.2 ± 4.1
FR	BL	7.34 ± 0.01	−1.08 ± 0.93	5.0 ± 0.7	60.2 ± 1.4	45.6 ± 2.1
	EOS	7.17 ± 0.02 *	−15.58 ± 0.73 *	37.6 ± 3.0 *	88.3 ± 4.2 *	31.3 ± 2.0 *
	EOO	7.21 ± 0.01 *^,#^	−11.85 ± 0.51 *^,#,†^	6.6 ± 0.8 ^†^	80.5 ± 5.7 *	37.3 ± 1.4

SH = sham-operated animals, RR = restricted reperfusion, FR = full reperfusion. Arterial blood gases (pH, base excess (ABEc), lactate, oxygen (pO_2_) and carbon dioxide (pCO_2_) partial pressure) were measured at baseline (BL), end of shock (EOS) and at the end of observation (EOO). n = 8 animals in each group. Data are presented as mean ± SEM, * indicates significance versus baseline, ^#^ indicates significance sham versus shock, ^†^ indicates significance EOS versus EOO, *p* < 0.05.
